# Tubal ectopic twin pregnancy of complete hydatidiform mole and coexisting embryo: A rare case report

**DOI:** 10.1097/MD.0000000000033922

**Published:** 2023-06-02

**Authors:** Yun Shen, Jianing Wang, Yawei Shao, Chanchan Gong, Ruiheng Zhao

**Affiliations:** a Department of Gynecology, Suzhou Ninth Hospital Affiliated to Soochow University, Suzhou, Jiangsu Province, China; b Department of Pathology, Suzhou Ninth Hospital Affiliated to Soochow University, Suzhou, Jiangsu Province, China.

**Keywords:** case report, complete hydatidiform mole, ectopic pregnancy

## Abstract

Ectopic twin gestation involving a complete hydatidiform mole (CHM) and coexisting embryo is an exceedingly rare occurrence.

**Patient concerns:**

In this report, we present the case of a 22-year-old female (gravida2, para 1) who was in her 8th week of gestation and presented with abdominal pain. Due to the detection of active bleeding and a ruptured right fallopian tube, the patient underwent a salpingectomy on the right side.

**Diagnosis:**

The patient was diagnosed with an ectopic twin gestation involving a CHM and coexisting embryo.

**Interventions:**

The patient was treated with right-side salpingectomy.

**Outcomes:**

The operation was successful and her recuperation was satisfactory.

**Lessons:**

In the management of ectopic pregnancy involving CHM, it is crucial to enhance the accuracy of preoperative diagnosis. Additionally, histopathological examination of the salpingectomy specimen and conceptus is definitely essential for accurate diagnosis and appropriate follow-up management of tubal pregnancies.

## 1. Introduction

Ectopic molar pregnancy is a rare occurrence, with an estimated incidence of approximately 1.5 per 1 million conceptions.^[1]^ As preoperative diagnosis can be difficult, postoperative histopathological diagnosis remains the primary diagnostic tool. Even rarer are cases of tubal complete hydatidiform moles (CHMs). In this report, we describe the case of a 22-year-old female who experienced an ectopic twin gestation of CHM and coexisting embryo in the same fallopian tube, which is an extremely uncommon event. To the best of our knowledge, no other cases of an ectopic twin gestation of CHM and coexisting embryo in the same fallopian tube have been reported. We believe that our case is unique, given that the gestation coexisted with a CHM mass on the same side of the fallopian tube. Herein, we provide a detailed description of the clinical and histologic findings relevant to this rare presentation. Written informed consent was obtained from the patient for the publication of this case report and accompanying images. Also, patient anonymity is preserved.

## 2. Case presentation

**Figure 1.**
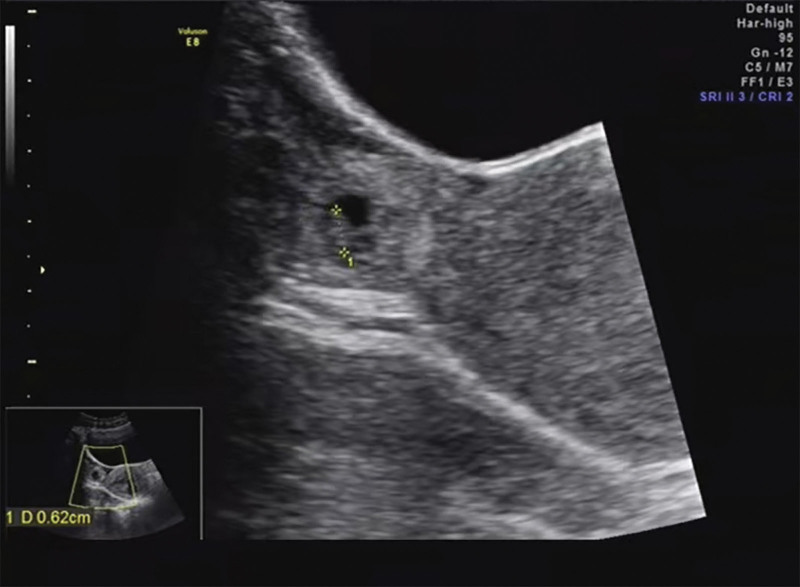
Pelvic ultrasound image revealed a heterogenous mass measured 22 × 21mm in the region of right adnexa containing a 14 × 9 × 11mm gestational sac (GS) along with a 6mm embryo with active heartbeats.

A 22-year-old female (gravida2, para 1) who conceived naturally, presented with abdominal pain in her 8th week of pregnancy. The patient had previously delivered a live infant 7 months earlier and was currently breastfeeding. Physical examination revealed tenderness in the right lower quadrant. Serum human chorionic gonadotropin (hCG) levels were measured and found to be 13,996 IU/L. Pelvic ultrasound revealed a heterogenous mass in the right adnexa measuring 22 × 21 mm, which contained a 14 × 9 × 11mm gestational sac (GS) and a 6mm embryo showing active heartbeats. Notably, no intrauterine GS was visualized on the ultrasound image (see Fig. 1).

The clinical diagnosis was ectopic pregnancy with a definite indication for surgery. Therefore, a decision was made to proceed with a diagnostic laparoscopy. Intraoperatively, the uterus was at normal volume, but the right fallopian tube was found to be thickened to approximately 3 cm in diameter and had ruptured in the interstitial part near the uterine connection. Given the detection of active bleeding and villus tissue, the decision was made to perform a right-sided salpingectomy, which had been agreed upon by the patient prior to surgery. Following the procedure, the patient experienced an uncomplicated recovery and was monitored using serial hCG and pelvic ultrasound examinations. Over time, her hCG levels gradually decreased to the normal range. Two weeks after surgery, an ultrasound examination showed no evidence of residual gestational trophoblastic disease.

**Figure 2.**
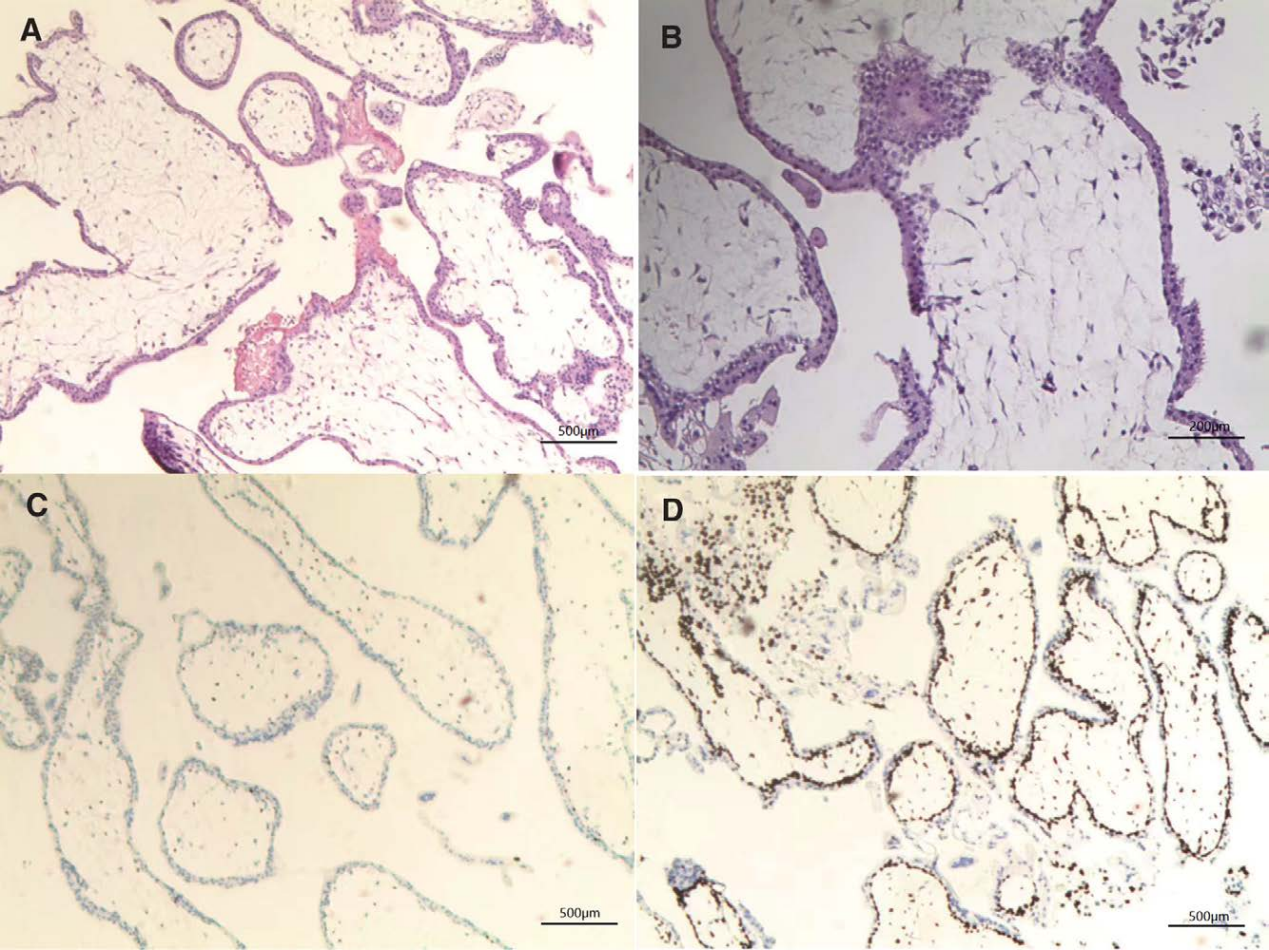
(A) Numerous large, edematous, and irregularly-shaped chorionic villi. (B) Circumferential trophoblastic proliferation. (C) Immunohistochemistry (IHC) study for p57 revealed absence of nuclear staining in the villous stromal and cytotrophoblastic cells. (D) IHC study for Ki-67 showed that Ki-67 proliferative index was positive 75%.

However, the histopathology report revealed an unusual result. Gross examination revealed both edematous and seemingly normal villi tissue. Upon microscopic examination, hematoxylin and eosin-stained slides showed numerous large, edematous, and irregularly-shaped chorionic villi (see Fig. 2A). Moreover, circumferential trophoblastic proliferation was also observed, which is consistent with the diagnosis of CHM (see Fig. 2B). Immunohistochemistry (IHC) staining for p57 and Ki-67 was carried out. As shown in Figure 2C, the villous stromal cells and cytotrophoblastic cells were negative for p57; however, adjacent intermediate trophoblasts served as a positive control. In addition, Ki-67 overexpression (with the Ki-67 proliferative index being 75%) was observed in the cytotrophoblasts (see Fig. 2D). Based on the combined morphologic findings and the p57 and Ki-67 IHC results, the final diagnosis was “tubal complete hydatidiform mole.” Given the previously mentioned pelvic ultrasound findings (a GS with embryonic heartbeat observed in the right adnexal area), a possible diagnosis of partial molar pregnancy should also be considered. To differentiate this from partial hydatidiform molar pregnancy, karyotype analysis was performed. The results confirmed a complete hydatidiform molar pregnancy, as indicated by the presence of the 46XX karyotype. Based on the aforementioned results, we have reached the bold conclusion that this is an extremely rare case of tubal ectopic twin pregnancy of CHM and coexisting embryo.

## 3. Discussion

Tubal molar pregnancy is an extremely rare event, with an estimated incidence of approximately 1.5 per 1 million conceptions.^[1]^ Completely hydatidiform mole coexistent with a normal fetus (CHMCF), it is an extremely rare condition that occurs from 1 in 20,000 to 1 in 100,000 pregnancies.^[2]^ A PUBMED search was conducted using the keywords “hydatidiform mole,” “ectopic molar pregnancy,” and “completely hydatidiform mole coexistent with a normal fetus.” However, our search did not reveal any cases of tubal pregnancy involving CHMCF. Therefore, we believe that this case represents the first report of such a presentation.

CHMCF is usually diagnosed in the second trimester of pregnancy when the molar placenta is well-documented by ultrasonography examination.^[2]^ However, it is well-known that tubal ectopic pregnancies should be diagnosed and treated as early as possible. Ultrasonography and β-hCG levels are not very helpful in differentiating between CHMCF and other ectopic pregnancies in the first trimester, due to the absence of the classic features previously described in the second trimester.^[3]^ Postoperative histopathological diagnosis remains the primary tool for diagnosing CHMCF. However, identifying early molar pregnancy can be challenging for many histopathologists.^[4–7]^ Early ectopic gestation sometimes presents florid trophoblastic proliferation so some pathologists may consider molar disease.^[8]^ The morphologic features of hydrops, scalloped villi, and stromal karyorrhexis can aid in diagnosing ectopic mole. Additionally, ancillary studies like IHC and molecular genotyping can significantly improve diagnostic accuracy.^[9]^ A negative result of p57 IHC supports the diagnosis of CHM by confirming the absence of a maternal allele. Banet et al^[10]^ demonstrated that p57 is almost always negative in the cytotrophoblastic cells and villous stromal cells of CHMs, while a positive p57 expression is found in almost all partial moles and nonmolar pregnancies. Ki-67 is also supposed as a useful adjunct for refining the diagnosis of CHM.^[11]^ Karyotype analysis is also of great importance in distinguishing between partial and complete molar pregnancies. It is important to note that CHMs are diploid and androgenetic in origin, with all 46 chromosomes derived from the father,^[12,13]^ while in partial hydatidiform mole, the paternal to maternal chromosome ratio is 2:1. In our case, the absence of p57 signal, high Ki-67 proliferative index, and diploid karyotype, confirmed the diagnosis of a CHM.

Owning to the scarcity of tubal CHM pregnancy cases, there are currently no established guidelines or expert consensus on diagnosis and treatment. Laparoscopy is typically utilized to treat ectopic pregnancies. Studies have shown that ruptured fallopian tubes are common in cases of tubal CHM pregnancy, thus salpingectomy is often the preferred treatment approach. When it comes to unruptured tubal pregnancies, without a preoperative diagnosis of tubal CHM pregnancy, salpingotomy may be considered as an alternative option. Pasic et al^[14]^ have suggested that salpingotomy should be the preferred surgical treatment option for most patients with unruptured tubal pregnancies. However, this approach may have hidden issues that could impact both short-term and long-term outcomes. In view of this situation, intraoperative rapid pathological diagnosis might be a breakthrough to avoid unnecessary injury.

In conclusion, we report a rare case of tubal pregnancy involving CHMCF. This serves as a reminder of the importance of improving preoperative diagnosis for effective and appropriate management of CHM ectopic pregnancy. Ongoing efforts are being made to enhance preoperative diagnostic capabilities. It cannot be overemphasized that histopathological examination of salpingectomy specimens and concepti in tubal pregnancies is essential for accurate diagnosis and appropriate follow-up management.

## Acknowledgments

The authors gratefully acknowledge the work of past and present members of our department.

## Author contributions

**Conceptualization:** Jianing Wang.

**Data curation:** Yun Shen, Chanchan Gong.

**Funding acquisition:** Jianing Wang.

**Investigation:** Yawei Shao.

**Project administration:** Ruiheng Zhao.

**Writing – review & editing:** Jianing Wang.**Writing – original draft:** Yun Shen.
